# A multi-faceted approach to promote knowledge translation platforms in eastern Mediterranean countries: climate for evidence-informed policy

**DOI:** 10.1186/1478-4505-10-15

**Published:** 2012-05-06

**Authors:** Fadi El-Jardali, Nour Ataya, Diana Jamal, Maha Jaafar

**Affiliations:** 1 Department of Health Management and Policy, American University of Beirut, PO Box 11–0236, Riad El Solh, Beirut, 1107 2020, Lebanon; 2 McMaster Health Forum, MML-417, 1280 Main St. West, Hamilton, ON, L8S 4L6, Canada; 3 Research, Advocacy and Public Policy-making, Issam Fares Institute for Public Policy and International Affairs, American University of Beirut, PO Box 11–0236, Riad El Solh, Beirut, 1107 2020, Lebanon

**Keywords:** Knowledge Translation, Evidence-Informed Policy, Eastern Mediterranean Countries

## Abstract

**Objectives:**

Limited work has been done to promote knowledge translation (KT) in the Eastern Mediterranean Region (EMR). The objectives of this study are to: 1.assess the climate for evidence use in policy; 2.explore views and practices about current processes and weaknesses of health policymaking; 3.identify priorities including short-term requirements for policy briefs; and 4.identify country-specific requirements for establishing KT platforms.

**Methods:**

Senior policymakers, stakeholders and researchers from Algeria, Bahrain, Egypt, Iran, Jordan, Lebanon, Oman, Sudan, Syria, Tunisia, and Yemen participated in this study. Questionnaires were used to assess the climate for use of evidence and identify windows of opportunity and requirements for policy briefs and for establishing KT platforms. Current processes and weaknesses of policymaking were appraised using case study scenarios. Closed-ended questions were analyzed descriptively. Qualitative data was analyzed using thematic analysis.

**Results:**

KT activities were not frequently undertaken by policymakers and researchers in EMR countries, research evidence about high priority policy issues was rarely made available, and interaction between policymakers and researchers was limited, and policymakers rarely identified or created places for utilizing research evidence in decision-making processes. Findings emphasized the complexity of policymaking. Donors, political regimes, economic goals and outdated laws were identified as key drivers. Lack of policymakers’ abilities to think strategically, constant need to make quick decisions, limited financial resources, and lack of competent and trained human resources were suggested as main weaknesses.

**Conclusion:**

Despite the complexity of policymaking processes in countries from this region, the absence of a structured process for decision making, and the limited engagement of policymakers and researchers in KT activities, there are windows of opportunity for moving towards more evidence informed policymaking.

## Introduction

During the last few years, global efforts have been projected towards promoting the use of research evidence in health policymaking [1-4]. The *Bamako Call to Action,* issued at the Global Ministerial Forum on Research for Health in November 2008, urged national governments and international funders to promote knowledge translation (KT), defined as a dynamic and iterative process that includes synthesis, dissemination, exchange and ethically-sound application of knowledge to improve the health of the population, provide more effective health services and products and strengthen the health care system [3,5]. It advocated evidence- informed health policymaking for improving health systems performance, which is defined as an approach to policy decisions that aims to ensure that decision making is well-informed by the best available research evidence. It is characterized by the systematic and transparent access to, and appraisal of, evidence as an input into the policymaking process [3,6]. The *Montreux Statement* at the First Global Symposium on Health Systems Research held in November 2010 reinforced the need for strengthening health systems research and enhancing its translation into policy [4]. In the Eastern Mediterranean Region (EMR), the Qatar Declaration (2008) urged ministries of health (MOH) and World Health Organization (WHO) to solicit research that addresses MOH needs and establish a center of excellence in health systems research and practice for information exchange [7]. More recently, WHO Eastern Mediterranean Regional Office (WHO EMRO) emphasized, in its strategic directions for research for health, the forceful implementation and expansion of research for health as a fundamental tool for health development and informing health policy [8].

In response to these repeated calls to action, *KT platforms*, which are partnerships between policymakers, researchers, civil society groups, and other key health system stakeholders, are being established worldwide by the WHO’s Evidence-Informed Policy Networks (EVIPNet), to facilitate the process of translating research evidence into policy and action [9]. Some of the activities supported by these KT platforms include production of research to address health policy priorities, systematic reviews, and policy briefs as well as the development of policy dialogues that bring different health actors together for strengthening evidence-informed policies [10-12]. So far, EVIPNet regional and country teams have been well-established in Africa, the Americas, and Asia [13]. In January 2009, EVIPNet was launched in the EMR, specifically in; Bahrain, Egypt, Iran, Jordan, Libya, Lebanon, Morocco, Oman, Pakistan, Sudan, Syria, Tunisia, and Yemen. EVIPNet EMR is a network coordinated by the regional office of the WHO and led by individuals at the country level, mostly those working in ministries of health in certain countries. For instance, there are established EVIPNet teams in Sudan and Jordan. It is worth noting that the authors of this paper are not actively engaged in EVIPNet activities in their country but they are researchers working at the department of health management and policy at the American University of Beirut, Lebanon.

Despite this, limited work has been done to date on KT in the EMR [14-17]. Much needs to be done to empower policymakers to access and use knowledge in policy and to develop KT country teams at the national level [8,13]. The EMR suffers from limited research capacity to generate health systems research and systematic reviews and to use it to improve health systems [16-18].

The objectives of this study are to: (1) assess the climate for the use of evidence in policy; (2) explore views and practices on the current processes and weaknesses of health policymaking; (3) identify priorities including short-term requirements for policy briefs; and (4) identify country-specific requirements and challenges for establishing KT platforms at the national level in selected EMR countries. This study is the first attempt in the EMR to provide essential knowledge that can be used to promote the climate for evidence-informed health policies and to develop KT platforms in EMR countries. This article is highly relevant and novel to the region, in the sense that it is the first of its kind to report on KT issues and challenges in the EMR. Findings from this study are potentially useful to countries from the region and in countries with similar characteristics and context. The article is particularly important in light of the newly released WHO Eastern Mediterranean Regional Office (WHO EMRO) strategic directions for research for health [8].

## Methods

**Figure 1:**
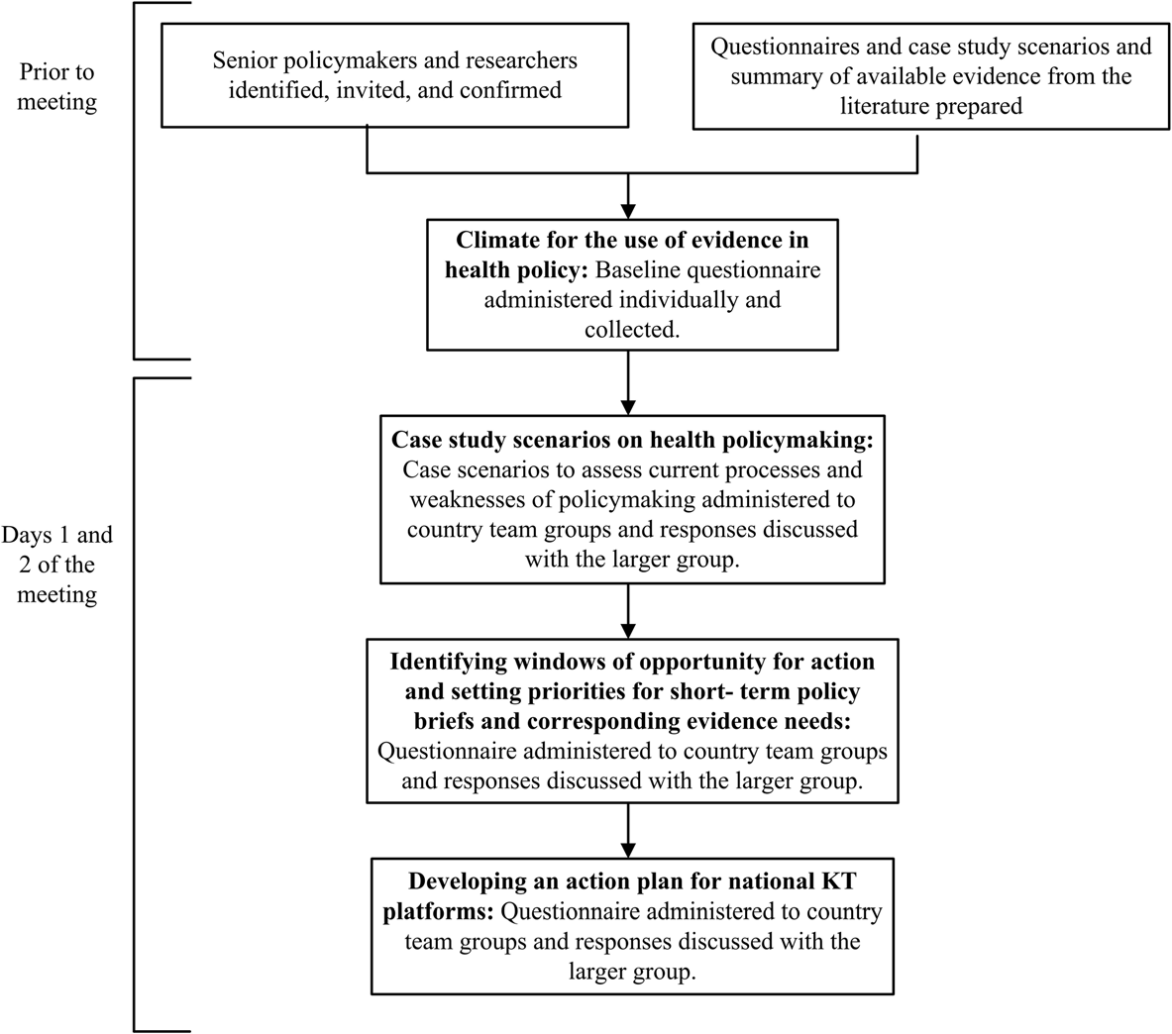
Multi- staged study approach.

This multi-staged study used a combination of quantitative and qualitative research techniques (Figure 1). We invited senior policymakers, stakeholders, and researchers from 11 countries to a meeting that was conducted in Beirut on December 2010. Study activities took place during two full days of the meeting. Countries included in this study were selected based on their interest and participation in EVIPNet EMR. These are: Algeria, Bahrain, Egypt, Iran, Jordan, Lebanon, Oman, Sudan, Syria, Tunisia, and Yemen. Purposive sampling of policymakers, stakeholders, and researchers was used to select participants. The sampling frame for the selection of respondents was adapted from a similar tool developed in Canada [19]. It included the following from each country:

· Policymakers at the national level including; senior officials from the MOH, civil society members, and health professional associations.

· Senior health systems researchers within national research institutions, universities, national governments.

Members of EVIPNet EMR from each country were asked to identify three to five potential participants based on the categories of the sampling frame.

To elicit views and practices about the current processes and weaknesses of health policymaking and identify policy brief priorities and country-specific requirements for establishing KT platforms, participants from the same country were grouped together during the two days of the meeting. Following each activity, country teams discussed their responses with other teams to identify cross-cutting issues and promote exchange. The American University of Beirut granted the study Institutional Review Board (IRB) approval. Study activities were conducted over four main stages (Figure 1):

1. Climate for the use of evidence in health policy

In the first stage of the study, and one day prior to the meeting, participants were asked to individually complete a baseline questionnaire to assess the general climate for KT. The questionnaire was adapted from a similar tool developed in Canada [19]. The validity of this tool was established through piloted-testing with policymakers, researchers, and stakeholders in various contexts. It was used in several countries, both from the region and beyond, to assess the climate for KT. These countries include Jordan, Sudan, Canada, Uganda, Zambia, Nigeria, Cameroon, and Malaysia (*Forthcoming*). The tool was translated to Arabic by a professional translator and back-translated to English; minimal differences were detected. The questionnaire included demographics and three quantitative scales. Demographics included country of residence, work, and years of experience. The first scale included seven items that assessed the availability of health research evidence about high-priority policy issues. The second scale included five items that assessed the strength of relationships among policymakers and researchers. The third scale included four items that assessed policymakers' capacity to support the use of health research evidence in health systems policymaking. Participants rated each item using a seven-point Likert scale (never, very rarely, rarely, occasionally, frequently, very frequently, always) (Additional file 1).

2. Case study scenarios on health policymaking

A problem-solving approach was used to assess current health policymaking processes and weaknesses. Five case studies on priority health policy issues that simulate real policymaking scenarios were developed by the research team in collaboration with KT experts. Case studies were prepared on five different topics: Primary Healthcare and Universal Accessibility and Equity; Public Private Partnerships; Improving Healthcare Quality through National Accreditation; Decisions in Case of Emergency: Example of H1N1 (commonly referred to as Swine Flu) as a Focusing Event; and Technology Assessment in the Case of Hyperbaric Oxygen Therapy (HBOT). The case study on Public Private Partnerships is provided as an example in Additional file 2.

The approach to case studies was guided by SUPPORT tools for evidence-informed decisions, which were written for people responsible for making decisions about health policies and programmes and for those who support them, and was adapted to the context of the region [10]. Each case study consisted of a problem statement and a summary of available evidence from the literature on the problem (including systematic reviews), potential policy options and implementation considerations. In addition, the case studies included specific questions that aimed to delineate different needs for informed decision-making, identify current processes and weaknesses of health policymaking, identify when and how policymakers search for evidence (explicit and tacit) and what they look for, and understand how knowledge is being used. Country teams were introduced to the case studies and asked to select one topic.

3. Identifying windows of opportunity for action and setting priorities for short-term policy briefs and corresponding evidence needs

A questionnaire consisting of two open-ended questions was used (Additional file 3). The first question asked country teams to identify windows of opportunity for action over the next six months to one year; for example, a political event or other events that may provide a window of opportunity to undertake action or introduce change [20]. The second question asked country teams to set priorities for policy briefs over the next six months to one year at the national level and the corresponding specific evidence required for each priority.

4. Developing an action plan for national KT platforms

Country teams were requested to complete a questionnaire to identify country-specific requirements and to develop a plan of action including challenges for establishing national KT platforms identify relevant target groups, define levels of functioning (local, provincial, national), identify location, core team, partnerships and linkages, needed support and commitment from stakeholders, and priorities for evidence-informed decision-making (Additional file 4). Teams were also asked to identify barriers to KT, as well as means to better produce, disseminate and use knowledge for decision-making.

### Data Analysis

Data from the baseline questionnaire, administered in the first stage on assessing the climate for the use of evidence in health policy, was entered on SPSS 18.0 and analyzed using quantitative data analysis. For closed-ended questions measured on seven-point Likert scales, we combined the responses very rarely and rarely as well as frequently and very frequently, resulting in a five-point scale (never, very rarely/rarely, occasionally, frequently/very frequently, and always). Descriptive analysis was performed for closed-ended questions (including demographics). Whereas, for the second, third, and fourth stages of the study, specifically “Case study scenarios on health policymaking”, “Identifying windows of opportunity for action and setting priorities for short-term policy briefs and corresponding evidence needs”, and “Developing an action plan for national KT platforms” open- ended questions were administered and were thus analyzed qualitatively. Thematic analysis was used for qualitative analysis. Responses were broken into similar concepts and ideas (open coding). Axial coding followed in which concepts were organized into themes [21]. Recurring themes and emerging patterns across countries were then analyzed. Analysis was conducted independently by two members of the research team. Disagreements were discussed until consensus was achieved. In addition, a note-taker was present with each team to document all deliberations that took place within country teams and across all teams. The notes taken by these individuals were used in the debrief each group provided at the end of each exercise. Notes were compiled and compared for accuracy against the notes of the moderator.

## Results

A high level of agreement was observed for the analysis conducted by the two members of the research team.

A total of 65 potential participants were identified and invited to participate. A total of 42 participated in the study (response rate of 64.6 %), 25 were policymakers and stakeholders and 17 were researchers.

### Climate for the use of evidence in health policy

A total of 27 respondents out of 42 (64.3 %) completed the survey. The average years of experience for respondents was 9.54 years (SD = 7.94). About half the respondents (51.9 %)indicated being policymakers within their national government and (Table 1).

**Table 1:** Baseline survey respondents’ profession

**Profession**	**N (%)**
Policymaker- national government	14 (51.9)
Researcher- university	4 (14.8)
Stakeholder- Staff/member of a civil society/non-governmental organization (NGO)	2 (7.4)
Researcher- national research institution	2 (7.4)
Researcher- Ministry of Health	1 (3.7)
Researcher- national research institute & university	1 (3.7)
Policymaker at national government & Researcher at Ministry of Health	1 (3.7)
Policymaker at healthcare institute & Stakeholder at civil society & Researcher at University	1 (3.7)
Other	1 (3.7)

Concerning the availability of evidence, only a quarter of respondents reported that research evidence on high priority policy issues was very frequently/frequently available to policymakers. In fact, more than half of respondents indicated that evidence on high priority policy issues was very rarely/rarely disseminated to policymakers, including articles or reports about primary research and policy briefs. Only two respondents reported that systematic reviews were frequently/very frequently disseminated to policymakers. Although the majority indicated that policymakers always had access to a personal computer with a functional internet connection, only some indicated that policymakers frequently/very frequently had access to research evidence through a searchable database or through a service operated by researchers and designed to respond to their questions in a timely way (Table 2).

**Table 2:** Baseline questionnaire results for assessing the climate for the use of evidence for policymaking in the region

	**Never**	**Very Rarely/Rarely**	**Occasionally**	**Frequently/Very Frequently**	**Always**
	**N (%)**	**N (%)**	**N (%)**	**N (%)**	**N (%)**
**1. How often was relevant research evidence about high-priority policy issues easily available to policymakers?**					
**a)** Copies of articles or reports about primary research on high-priority policy issues were widely disseminated to policymakers working on these issues.	0 (0.0%)	14 (51.9%)	4 (14.8%)	8 (29.6%)	1 (3.7%)
**b)**Systematic reviews of the research literature on high-priority policy issues were widely disseminated to policymakers working on these issues.	7 (26.9%)	10 (38.5%)	6 (23.1%)	2 (7.7%)	1 (3.8%)
**c)** Policy briefs that described research evidence about a high-priority problem, options for addressing the problem, and key implementation considerations were widely disseminated to policymakers working on these issues.	2 (7.7%)	17 (65.4%)	3 (11.5%)	4 (15.4%)	0 (0.0%)
**d)** Policymakers had access to a personal computer with a functional internet connection.	0 (0.0%)	0 (0.0%)	3 (11.5%)	7 (26.9%)	16 (61.5%)
**e)** Policymakers had access to research evidence on high-priority policy issues through a searchable database focused on these issues.	2 (7.4%)	12 (44.4%)	4 (14.8%)	8 (29.6%)	1 (3.7%)
**f)** Policymakers had access to research evidence on high-priority policy issues through a service operated by researchers and designed to respond in a timely way to questions about these issues.	5 (18.5%)	11 (40.7%)	4 (14.8%)	6 (22.2%)	1 (3.7%)
**g)** Research evidence concerning high-priority policy issues was available to policymakers.	1 (3.7%)	10 (37%)	9 (33.3%)	7 (25.9%)	0 (0.0%)
**2. How often did policymakers and researchers interact in the following ways?**					
**a)** Policymakers interacted with researchers as part of a priority-setting process to identify high-priority policy issues for which primary research and systematic reviews were needed.	1 (3.7%)	15 (55.6%)	6 (22.2%)	5 (18.5%)	0 (0.0%)
**b)** Policymakers interacted with researchers as part of the process of conducting primary research or systematic reviews about high-priority policy issues.	0 (0.0%)	12 (46.2%)	7 (26.9%)	7 (26.9%)	0 (0.0%)
**c)** Policymakers interacted with researchers to obtain assistance with finding and using research evidence about high-priority policy issues.	2 (7.7%)	8 (30.8%)	7 (26.9%)	9 (34.6%)	0 (0.0%)
**d)** Policymakers interacted with researchers through targeted efforts to support research use in policymaking (i.e., a rapid-response service or policy dialogues).	2 (8.3%)	17 (70.8%)	1 (4.2%)	4 (16.7%)	0 (0.0%)
**e)** Policymakers interacted with researchers on an informal basis (i.e., through membership on committees, attendance at meetings, personal conversations).	0 (0.0%)	6 (22.2%)	9 (33.3%)	8 (29.6%)	4 (14.8%)
**3. How often did policymakers develop and demonstrate their capacity to find and use health research evidence in health systems policymaking?**					
**a)** Policymakers participated in training to develop their capacity to find and use research evidence about high-priority policy issues.	4 (15.4%)	11 (42.3%)	6 (23.1%)	5 (19.2%)	0 (0.0%)
**b)** Policymakers acquired research evidence on high-priority policy issues.	1 (3.8%)	12 (46.2%)	6 (23.1%)	7 (26.9%)	0 (0.0%)
**c)** Policymakers assessed the quality and local applicability of research evidence on high-priority policy issues.	6 (23.1%)	11 (42.3%)	5 (19.2%)	4 (15.4%)	0 (0.0%)
**d)** Policymakers identified or created places for research evidence in decision-making processes.	2 (7.7%)	14 (53.8%)	6 (23.1%)	4 (15.4%)	0 (0.0%)

When asked about relationships between policymakers and researchers, half of the respondents indicated that policymakers very rarely/rarely interact with researchers as part of priority-setting processes or for conducting primary research or systematic reviews. Furthermore, the majority reported that policymakers very rarely/rarely interacted with researchers through targeted efforts to support research use in policymaking. One-third reported that policymakers frequently/very frequently interacted with researchers to obtain assistance with finding and using research evidence and occasionally interacted with researchers on an informal basis (Table 2).

Furthermore, respondents reported that policymakers very rarely/rarely identified or created places for utilizing research evidence in decision-making acquired research evidence on high priority policy issues, or participated in training to develop their capacity to find and use evidence and assess its quality and local applicability (Table 2).

### Current processes and weaknesses of health policymaking

The case scenarios elucidated current processes for policymaking, and sources of knowledge (both explicit and tacit). Participants emphasized that not all information is accessible and used when making decisions, as one researcher stated, *“In our country [certain] information [for policymaking] is not made public. There is a need to work on a law to promote the exchange of information between producers and users”*. Participants also stated that local evidence from health systems and policy research (HSPR) is insufficient in the region and the nature of the available information is often inadequate for decision-making. For example, participants reported the need for local evidence on the HRH needs, patient satisfaction, and means to implement national health insurance schemes in their own countries. All country teams agreed that information on the cost-effectiveness of implementing an intervention was essential for decision-making on the various topics of the case studies. Furthermore, all country teams emphasized the need for information on the available human resources for health (HRH) in their countries, specifically the number of available human resources, their capacity, and incentives required to implement specific interventions. Other essential information for decision-making varied across different case studies. For example, country teams discussing Public Private Partnerships and strategies to improve access to Primary Healthcare reported that they mostly needed information on the quality of services. Additionally, country teams working on implementing strategies to improve access to Primary Healthcare indicated that information on the geographical distribution, health-seeking behavior, as well as needs and health utilization of the population was essential for decision-making. Country teams stated that most of the essential information for decision-making was available with various MOH departments, national registries, research studies, and international reports. Information types and corresponding sources as listed by country teams are presented in Table 3. Participants also agreed that the MOH should first implement an audit to assess the available information pool including mapping tacit knowledge and grey literature. As one researcher stated, *“It is important to use knowledge [from] those with the know-how. The challenge is how to tap into tacit knowledge, this is an issue we are facing”.* Upon presenting participants with the summary of available evidence on the case studies, most stated that the provided evidence lacked information on cost-effectiveness, monitoring and evaluation, quality of services and patient satisfaction as well as context-specific information.

**Table 3:** Type and sources of data/information/knowledge used for decision-making in the region

**Type**	**Source**
Cost- effectiveness	Systematic reviews, literature reviews, MOH, Professional associations, Committees
Human resources (needs and supply, incentives, capacities)	Professional associations
Infrastructure (availability of equipment, maintenance)	Private sector, MOH
Population status and needs, future projection of the population	National registry, MOH information system
Quality of services	MOH, Accreditation surveys, Research, Client satisfaction
Existing policies and procedures	Healthcare institutions, regulations
Evaluation of outcomes and impact	International reports
Information on patient satisfaction	Research, Scientific committees
Legal information: legal text and legislations	Legal text, constitution
**Other:** Efficiency, Feasibility analysis, Situational analysis, Types and range of services	**Other:** Qualitative studies, Subject experts & researchers, National and International experience, WHO Reports, Accreditation, Demographic and health surveys, National Health Accounts, Ministry of Finance, Health Expenditure and Utilization Surveys

In response to a question on the current processes for decision-making on topics of the case studies, the MOH was identified as the main decision-maker by four country teams; while in other countries, advisory panels were involved in the decision-making process. Furthermore, participants across countries identified several key drivers of health policymaking in the EMR. Donors were mentioned as one of the main drivers of the policymaking process. As a policymaker explained, “*Donors are directing us in certain directions… Influential people in the country have their own projects and will push them forward; which affects policymaking*”. Political regimes, economic goals, as well as laws and regulations that govern health systems were also cited as strong drivers of policymaking. As another policymaker stated “*It is difficult to change existing laws and regulations, even if they are outdated, and often it is difficult to propose new laws and regulations, so policymakers try to work around existing laws and regulations by doing marginal adjustments to the status quo*”. Furthermore, discussions with participants emphasized the complexity of the health policymaking process. Participants agreed that current health policymaking processes are not structured and the evaluation of these processes does not exist. For most country teams, decision-making is not based on evidence, as a policymaker explained “*Sometimes [policymakers] are reactive instead of proactive. Although they hire technical people, they do not take their opinion into consideration when making decisions*”.

In terms of the weaknesses of current health policymaking, country teams reported lack of policymakers’ abilities to think strategically and develop policies, financial constraints, and lack of competent and skilled human resources in policymaking institutions. The constant need to make quick decisions regardless of the availability of evidence was another frequently mentioned weakness of health policymaking, as one policymaker stated “*Time is an important factor in the use of evidence in policymaking*”.

### Identifying windows of opportunity for action

When asked to identify windows of opportunity for action over the next six months to one year, one recurring theme was the development of new national strategic plans. Furthermore, most country teams reported that changes in government (at the level of the Minister or the Ministry) would allow the introduction of KT activities. Implementing national health surveys was also frequently mentioned as a window of opportunity for action. Country teams from Algeria, Lebanon, Syria, and Yemen indicated an intent to implement national health accounts and household surveys in the future, which will enable usage of generated evidence in strengthening their health policies. The implementation of new programs was also identified as an opportunity for action in some countries. Bahrain country team indicated that a draft on compulsory health insurance was in parliament for discussion and would provide an opportunity for using research evidence for formulating this policy. Furthermore, during large group discussions, many participants identified the strategic directions for scaling up research for health in the EMR by WHO EMRO [8], as an important opportunity for action, as it focuses on promoting KT and use of evidence in health policies.

### Setting priorities for short-term policy briefs and corresponding evidence needs

Regarding priority topics for short-term policy briefs, the most frequent priorities across countries were related to national health insurance and universal health coverage. Most country teams needed evidence about means to implement effective national insurance schemes. Healthcare quality was also a commonly identified priority for short-term policy briefs. Most country teams needed evidence on ways to improve quality of service delivery. Another frequently identified priority for policy briefs was HRH. Specific evidence needs for informing HRH priorities include effective mechanisms for the HRH management such as training, increasing salaries, and performance-based payments. Some country teams also identified financial resources and allocation as a priority and required evidence on how to allocate health budgets and spending based on population needs and priorities (Table 4).

**Table 4:** Priorities for short- term policy briefs and specific evidence needs*

**Priority topics for short-term policy briefs**	**Specific evidence needs**
**1. National health insurance and universal health coverage**	Means to implement effective national health insurance schemes
	Accurate estimation of health utilization and expenditure from the private and public sectors including out-of-pocket expenditure.
**2. Healthcare quality**	Ways to improve the quality of service delivery including Accreditation strategies.
	Ways that can enable the use of performance indicators to improve quality.
	Information on patient satisfaction.
	Information on performance indicators (clinical, human resources productivity and performance).
**3. Human resources for health**	Effective incentives mechanisms for the management of healthcare workforce including training, salaries, and performance-based payments.
	Means to reduce disparities among the distribution of healthcare workers in order to meet national population needs.
**4. Financial resources and allocation: budgeting and government health expenditure**	Means to allocate health budgets and spending based on population needs and priorities.
	Information on the supply system to hospitals and healthcare centers.
**5. Maternal and child mortality**	Ways to reduce maternal and infant mortality.
**6. Management of healthcare facilities**	Ways to better manage healthcare facilities by the state.
**7. Building national information systems**	Means to develop national information systems for the population.
**8. Non-communicable diseases with a focus on cancer and diabetes**	Means to improve accessibility and services of programs for non-communicable diseases.

*Themes are reported in order of their recurrence across countries, from the most frequently reported (common across six countries) to those less frequently reported (common across two countries).

### Country-specific requirements and plans including challenges for establishing KT platforms

Most country teams emphasized the concept of networking and partnerships among stakeholders for their KT platforms. They intended to develop KT platforms at the national level and specifically at the MOH. They also indicated that the core team for their KT platforms will mostly be composed of policymakers, researchers, and stakeholders (NGO’s, professional associations, etc.) (Table 5). Most reported that the support and advocacy of policymakers was indispensable for establishing KT platforms (Table 5).

**Table 5:** Country action plans

**Action plan item**	**Most common responses across country teams**
**Concept**	▪ Informed decision- making and sharing of knowledge
	▪ Build partnerships and networking between all stakeholders to advocate for better health
**Target groups**	▪ Health professionals and professional associations
	▪ NGOs
	▪ Policymakers at Government and MOH
**Level**	▪ National
**Location**	▪ MOH
**Core team**	▪ MOH
	▪ Researchers and universities
	▪ NGOs and civil societies
	▪ Health professionals and professional associations
	▪ International organizations
**Partnerships and**	▪ MOH
**linkages**	▪ Universities and research institutions
	▪ NGOs and civil societies
	▪ International organizations
	▪ Donors
**Stakeholders support**	▪ Advocacy of policymakers
	▪ Financial resources
	▪ Accesses to relevant information and research (including tacit knowledge)
	▪ Technical and capacity-building on KT activities
**Financial support**	▪ International
	▪ National
**Barriers**	▪ Lack of communication between researchers and policymakers
	▪ Lack of financial resources
	▪ Lack of policy-relevant research (e.g. systematic reviews)
	▪ Lack of documentation of experiences and lack of mechanisms to utilize tacit knowledge
	▪ Subjective nature of decision-making processes
	▪ Short life of the MOH
	▪ Research messages are not clear for policymakers
**Plans**	▪ Policy briefs
	▪ Policy dialogues
	▪ Building relationships with the media
	▪ Publications
	▪ Working teams for knowledge creation and translation
	▪ Websites, emails, and newsletters.
	▪ National/regional conferences/networking, workshops and seminars.
**M&E**	▪ Set performance indicators
	▪ Timely policy briefs and evaluation of impact.

Key barriers to KT activities included lack of communication between researchers and policymakers, lack of financial resources, lack of policy-relevant research particularly systematic reviews, lack of documentation of experiences, lack of mechanisms to utilize tacit knowledge, as well as subjective nature of decision-making processes (Table 5). Discussion with the larger group also revealed additional barriers including weak MOH infrastructure, lack of/shortage in governance, political sensitivity of findings, low motivation and incentives for KT and for utilizing evidence, lack of capacity to use research, and lack of synchronization between policy and research priorities. The most commonly planned KT activities were developing policy briefs and policy dialogues.

## Discussion

### Principal findings

Study findings show that KT activities are not frequently undertaken by policymakers and researchers in EMR countries. Most respondents indicated that research evidence about high priority policy issues was rarely made available to policymakers, interaction between policymakers and researchers was limited and mostly informal, and that policymakers rarely identified or created places for utilizing research evidence in decision-making. Furthermore, findings from the case study scenarios emphasize the complexity of the policymaking process in countries and the absence of a structured process for decision-making, as well as the limited information available and limited access and use of tacit knowledge in policymaking.

Several windows of opportunity for action and change were identified including development of new strategic plans, changes in governments, reform initiatives and implementation of national health surveys and new programs. The most commonly identified priority topics for policy briefs were related to national health insurance and universal health coverage, healthcare quality, human resources for health, and financial resources and allocation. In terms of the requirements to establish KT platforms in countries, respondents emphasized the need for support and advocacy of policymakers, financial resources from both national and international bodies, technical and capacity-building on KT activities, and access to relevant information and research.

### Strengths and limitations

To our knowledge, this is the first study to bring together senior policymakers, stakeholders and researchers from EMR countries to explore issues related to strengthening health policymaking through KT. It is one of the first to identify priorities for short-term requirements for policy briefs and more importantly the first in the region to assess the climate for the use of evidence, the processes and weaknesses of health policymaking and the requirements for establishing KT platforms.

Our methodology has several strengths. First, grouping researchers and policymakers from the same country together encouraged dialogue and helped obtain a holistic perspective on health policymaking, barriers and needs for establishing KT platforms, and facilitated the formation of core country teams for KT platforms. Second, we used a combination of qualitative and quantitative research techniques to gain a more in-depth analysis of the views and practices of participants. We also used case study scenarios as techniques to simulate real policymaking situations and practices. Third, study activities helped obtain commitment from key stakeholders such as WHO EMRO to support countries in establishing KT platforms and mobilizing resources to promote KT in the region. WHO EMRO’s support for strengthening KT in the region was recently highlighted in its strategic directions, which called for promoting the concept of EVIPNet in the region and enhancing policy advocacy for needed buy-in and support [8]. Finally, our approach can be replicated in other national and regional contexts as an effective strategy for initiating, promoting and strengthening KT. Our study resulted in the development of templates to guide countries in identifying windows of opportunity, priority topics for policy briefs, and to develop country action plans for KT platforms (Additional file 2, Additional file 3 and Additional file 4). These can be utilized for conducting future capacity-building sessions and for gathering more information on policymaking processes.

Our study has a few limitations. A relatively small number of policymakers and researchers were selected to participate. Furthermore, respondents were purposefully (rather than randomly) selected; therefore, the findings might not be representative of all stakeholders. However, since participants represented senior positions it can be safely assumed that they are well-informed on KT activities in their countries. Furthermore, we have no information about the activities, outputs and outcomes of the implementation of the country KT action plans. In one year’s time, we plan to re-administer the survey to re-assess the climate for the use of evidence and compare with baseline data to identify shifts in how evidence is being used.

### Findings in relation to other studies

Some of the identified priorities for policy briefs and corresponding evidence needs, specifically relating to health financing, national health insurance and universal health coverage, and HRH coincide with those reported in a previous priority-setting exercise from the region [16]. This reflects limited efforts in the region to produce evidence to inform these policy-relevant priorities [9,17,22]. The complexity of the policymaking process and barriers to KT further corroborate those previously reported from the region [8,14,15]. Difficulty accessing information can be attributed to lack of capacity [14,15] or restricted access in some countries in the region. Furthermore, country teams’ needs for establishing KT platforms echoed those previously reported by policymakers and researchers in the region [14,15]. This emphasizes the urgency of mobilizing efforts and providing resources for developing and expanding KT platforms at the national level. As one policymaker stated *“We hope this momentum will carry on, giving more attention to country-level capacity building on evidence-based policymaking and KT to strengthen health systems performance and ultimately improve the health status of the population”.*

### Implications for policy and research

This study provides baseline assessment of KT in some EMR countries, and the methodology can be used in future studies to guide the establishment of KT platforms in other countries. Furthermore, our study provides priority themes for short-term policy briefs and specific research questions and evidence needs. Our findings can provide insights and guidance on strengthening the decision making process in health care in EMR countries.

Our findings can also inform and direct future plans and activities for key stakeholder organizations in the region including WHO EMRO, and Middle East and North Africa Health Policy Forum, and Gulf Cooperation Council. Future research is needed in countries to evaluate implemented KT strategies including policy briefs and policy dialogues [11,12].

## Conclusion

The complexity of the policymaking process in countries from the region, the absence of a structured process for decision-making, and the limited engagement of policymakers and researcher from the region in KT activities indicate that the path of promoting evidence-informed policies is a long and winding road. Still, there are substantial windows of opportunity for action towards evidence-informed policymaking through the development of new health system strategic plans, and reform initiatives in the region. Strengthening evidence- informed policies depends on a myriad of factors: availability of effective guides and providing technical support to KT country teams; leadership commitment, political engagement and shared ownership at the country-level, strengthened policymaking institutions (i.e. MOH), and conducting ongoing monitoring and evaluation of the use of evidence into policy. Findings from this study can guide efforts and direct resources towards strengthening health policymaking in countries through KT. In addition, study findings are useful for countries that host or are planning to host KT platforms.

As changes unfold in the EMR, one can only wonder about their implications for the development of health systems. Much analysis is now needed on understanding the underlying transformations and their respective health policy implications including the way evidence is translated into policy decisions.

## Abbreviations

EMR, Eastern Mediterranean Region; EVIPNeT, Evidence-Informed Policy Networks; HBOT, Hyperbaric Oxygen Therapy; HRH, Human Resources for Health; HSPR, Health Policy and Systems Research; IDRC, International Development Research Centre; IRB, Institutional Review Board; KT, Knowledge Translation; MOH, Ministry of Health; NGO, Non-Governmental Organization; WHO EMRO, WHO Eastern Mediterranean Regional Office; WHO, World Health Organization.

## Competing interests

The author(s) declare that they have no competing interests.

## Authors’ contributions

All authors meet criteria for authorship. All authors approved this version of the article for submission. FE-J contributed to the conception, design, and interpretation of the data as well as to drafting and critically revising the article. NA, DJ and MJ contributed to acquisition of data, analysis, interpretation as well as drafting and critically revising the article.

## Supplementary Material

Additional file 1 Baseline Survey. Click here for file

Additional file 2 Public Private Partnerships. Click here for file

Additional file 3 Identifying windows of opportunity for action and setting priorities for short-term policy briefs and corresponding evidence needs. Click here for file

Additional file 4 Developing an action plan for national KT platforms. Click here for file
