# Supplementary material for: A multi-faceted approach to promote knowledge translation platforms in eastern Mediterranean countries: climate for evidence-informed policy
Source: Health Res Policy Syst. 2012 May 6;10:15. doi: 10.1186/1478-4505-10-15 (PMC3445832; doi:10.1186/1478-4505-10-15)
Supplement: Additional file 1 — Baseline Survey. [file 1478-4505-10-15-S1.doc]

**Supplemental Material 1**

**Baseline Survey**

Several questions make reference to "stakeholders". The term "stakeholders" includes: staff or members of civil society groups; staff or members of health professional associations or groups; and staff of donor agencies (e.g., European Community) or international organizations (e.g., World Health Organization).

A number of questions offer "Don't know" as a response option which should be selected **only** if you feel that you do not have sufficient information or knowledge to form a view.

**Section 1: Views about evidence availability, interactions among policymakers and researchers, and policymakers’ capacity to find and use research evidence**

Please indicate how often, in your view, the following situations occurred in your country over the last two years.

1. How often was relevant research evidence about high-priority policy issues easily available to policymakers?

| **Never** | | **Very rarely** | **Rarely** | **Occasionally** | **Frequently** | | **Very frequently** | | | | **Always** | | |
| --- | --- | --- | --- | --- | --- | --- | --- | --- | --- | --- | --- | --- | --- |
| **1** | | **2** | **3** | **4** | **5** | | **6** | | | | **7** | | |
|  | Copies of articles or reports about primary research on high-priority policy issues were widely disseminated to policymakers working on these issues. | | | | | 1 | 2 | 3 | 4 | 5 | 6 | 7 | Don't  know |
|  | Systematic reviews of the research literature on high-priority policy issues were widely disseminated to policymakers working on these issues. | | | | | 1 | 2 | 3 | 4 | 5 | 6 | 7 | Don't  know |
|  | Policy briefs that described research evidence about a high-priority problem, options for addressing the problem, and key implementation considerations were widely disseminated to policymakers working on these issues. | | | | | 1 | 2 | 3 | 4 | 5 | 6 | 7 | Don't  know |
|  | Policymakers had access to a personal computer with a functional internet connection. | | | | | 1 | 2 | 3 | 4 | 5 | 6 | 7 | Don't  know |
|  | Policymakers had access to research evidence on high-priority policy issues through a searchable database focused on these issues. | | | | | 1 | 2 | 3 | 4 | 5 | 6 | 7 | Don't  know |
|  | Policymakers had access to research evidence on high-priority policy issues through a service operated by researchers and designed to respond in a timely way to questions about these issues. | | | | | 1 | 2 | 3 | 4 | 5 | 6 | 7 | Don't  know |
| g | Research evidence concerning high-priority policy issues was available to policymakers. | | | | | 1 | 2 | 3 | 4 | 5 | 6 | 7 | Don't  know |

2) How often did policymakers and researchers interact in the following ways?

| **Never** | | **Very rarely** | **Rarely** | **Occasionally** | **Frequently** | | **Very frequently** | | | | **Always** | | |
| --- | --- | --- | --- | --- | --- | --- | --- | --- | --- | --- | --- | --- | --- |
| **1** | | **2** | **3** | **4** | **5** | | **6** | | | | **7** | | |
|  | Policymakers interacted with researchers as part of a priority-setting process to identify high-priority policy issues for which primary research and systematic reviews were needed. | | | | | 1 | 2 | 3 | 4 | 5 | 6 | 7 | Don't  know |
|  | Policymakers interacted with researchers as part of the process of ***conducting*** primary research or systematic reviews about high-priority policy issues. | | | | | 1 | 2 | 3 | 4 | 5 | 6 | 7 | Don't  know |
|  | Policymakers interacted with researchers to obtain assistance with finding and using research evidence about high-priority policy issues. | | | | | 1 | 2 | 3 | 4 | 5 | 6 | 7 | Don't  know |
|  | Policymakers interacted with researchers through targeted efforts to support research use in policymaking (i.e., a rapid-response service or policy dialogues). | | | | | 1 | 2 | 3 | 4 | 5 | 6 | 7 | Don't  know |
|  | Policymakers interacted with researchers on an informal basis (i.e., through membership on committees, attendance at meetings, personal conversations). | | | | | 1 | 2 | 3 | 4 | 5 | 6 | 7 | Don't  know |

3) How often did policymakers develop and demonstrate their capacity to find and use health research evidence in health systems policymaking?

| **Never** | | **Very rarely** | **Rarely** | **Occasionally** | **Frequently** | | **Very frequently** | | | | **Always** | | |
| --- | --- | --- | --- | --- | --- | --- | --- | --- | --- | --- | --- | --- | --- |
| **1** | | **2** | **3** | **4** | **5** | | **6** | | | | **7** | | |
|  | Policymakers participated in training to develop their capacity to find and use research evidence about high-priority policy issues. | | | | | 1 | 2 | 3 | 4 | 5 | 6 | 7 | Don't  know |
|  | Policymakers acquired research evidence on high-priority policy issues. | | | | | 1 | 2 | 3 | 4 | 5 | 6 | 7 | Don't  know |
|  | Policymakers assessed the quality and local applicability of research evidence on high-priority policy issues. | | | | | 1 | 2 | 3 | 4 | 5 | 6 | 7 | Don't  know |
|  | Policymakers identified or created places for research evidence in decision-making processes. | | | | | 1 | 2 | 3 | 4 | 5 | 6 | 7 | Don't  know |

**Section 2: Role and background**

4) Please name your country:________________________________________

1. I am a ….(tick (√ ) single most appropriate role category):

| **Broad role category** | **Specific role category** | **Tick**  **(√)**  **single most appropriate** |
| --- | --- | --- |
| Policymaker | Public policymaker (i.e., elected official, political staff, or civil servant) in the national government |  |
|  | Public policymaker (i.e., elected official, political staff, or civil servant) in a sub-national government (e.g., province/state or a district if the latter has independent policymaking authority) |  |
|  | Manager in a district/region (if it does not have independent public policymaking authority) |  |
|  | Manager in a healthcare institution (e.g., hospital) |  |
| Stakeholder | Staff/member of a civil society group/ non-governmental organization NGO |  |
|  | Staff/member of a health professional association or group |  |
|  | Staff of a donor agency (e.g., European Community) or international organization (e.g., World Health Organization) |  |
|  | Representative of another stakeholder group |  |
| Researcher | Researcher in the Ministry of Health |  |
|  | Researcher in a national research institution |  |
|  | Researcher in a university |  |
|  | Researcher in another institution |  |
| Other- please specify |  |  |

1. I have been working in my current position for _____ years.
2. If you identified yourself as a policymaker, stakeholder, or "other," please indicate if you have training and/or extensive experience as a researcher (circle one): Yes / No
3. If you identified yourself as a researcher, stakeholder, or "other," please indicate if you have experience as a policymaker (circle one): Yes / No

**Thank you.**

**Additional thoughts (Optional)**

Do you have any comments regarding issues raised in particular questions? Kindly identify the question by number and add your comments in the space provided below.

_______________________________________________________________________________________ _________________________________________________________________________________________________________________________________________________________________________________________________________________________________________________________________________________________________________________________________________________________________________________________________________________________________________________________________________________________________________________________________________________________________________________________________________________________________________________________________________________________________________________________________________________________________________________________________________________________________________________________________________________________________________________________________________________________________________________________________________________________________________________________________________________________________________________________________________________________________________________________________________________________________________________________________________________________________________________________________________________________________________________________________________________________________________________________________________________________________________________________________________________________________________________________________________
