# Supplementary material for: A multi-faceted approach to promote knowledge translation platforms in eastern Mediterranean countries: climate for evidence-informed policy
Source: Health Res Policy Syst. 2012 May 6;10:15. doi: 10.1186/1478-4505-10-15 (PMC3445832; doi:10.1186/1478-4505-10-15)
Supplement: Additional file 2 — Public Private Partnerships. [file 1478-4505-10-15-S2.doc]

**Supplemental Material 2**

**Case Study**

**Public Private Partnerships**

Public private partnerships (PPP’s), in which the state delegates its responsibility to another actor to act on its behalf, have become increasingly common as a tool to improve the performance of the health system. The Ministry of Health is considering public private partnerships as a reform tool and should decide on the appropriateness of this tool and key implementation considerations.

**Please answer the following questions regarding this scenario and prepare yourself for discussion with other groups who have other scenarios.**

1. Which kind of data/ information/ knowledge is (are) essential for decision- making in this regard? Prepare a list.
2. What sources of data/ information/ knowledge should be considered for this purpose? Provide a table of these sources crossing with kind of data/ information/ knowledge.
3. The facilitator will give you the summary of data for this scenario. Is this information relevant to you? What is still missing to make a well- informed decision?
4. Suppose this is a real case in your country, what is the current process for decision- making within the country?
5. Is the existing knowledge about this issue appropriately used for policymaking?
6. In general, what are the current weaknesses of the existing policymaking process?

**Summary of Data**

In **Public private partnerships (PPPs)** the private entity is not paid by the users of the service but by the public entity that entered into the contract with it. Private actors take on the financing, design, construction, and maintenance of a health care facility while the public entity will be responsible for the health care function of the facility. Benefits, harms, examples and implementation considerations are summarized below.

| **Benefits** | **Harms** | **Examples** | **Implementation Considerations** |
| --- | --- | --- | --- |
| - No withdrawal of the state, but a change in its level of involvement. - The state is the owner of the infrastructure and is thus better able to reconsider the delegation of management. | This approach is still experimental. PPPs were criticized for leading to greater privatization in the health system. | - The state delegated the management of public hospitals to private firms in South Africa, Benin, and Mali. | - Policymakers should take into account specific legal forms depending on their national context. |
| - Provision of primary health care services to geographical areas that are inadequately served. - It can help young physicians set up their practice in rural areas where health facilities are lacking in exchange for their provision of primary health care as defined by the national health policy. - Contracting private medicine in rural areas is a possible solution when the conventional approach using health facilities is not possible for financial reasons. |  | - In Bangladesh, Cambodia, Madagascar and Mali the state contracted private firms, NGOs, and physicians for the provision of primary health care services in geographical areas that are considered inadequately served. | - Responsibility is not transferred in full. Links still exist between the state and these entities, which are difficult to regulate through a conventional hierarchical relationship. - The state should ensure that the contractee complies with the public service mission through the contract. |
| - PPPs decrease the burden on public financing and relief the state from the need to find funds to make its investments. |  |  |

There are no clear implementation considerations and design features for the effective implementation of public private partnerships in countries from the Eastern Mediterranean Region (EMR). Evidence does not assess the effectiveness of these contractual arrangements on the health system. The quality of the evidence is low; all reports are case studies.
