# Supplementary material for: A multi-faceted approach to promote knowledge translation platforms in eastern Mediterranean countries: climate for evidence-informed policy
Source: Health Res Policy Syst. 2012 May 6;10:15. doi: 10.1186/1478-4505-10-15 (PMC3445832; doi:10.1186/1478-4505-10-15)
Supplement: Additional file 3 — Identifying windows of opportunity for action and setting priorities for short-term policy briefs and corresponding evidence needs. [file 1478-4505-10-15-S3.doc]

**Supplemental Material 3**

**Identifying windows of opportunity for action and setting priorities for short-term policy briefs and corresponding evidence needs**

In this session, you are requested to identify priority topics for short- term policy briefs over the next ***6 months to 1 year*** at the national level. You are also required to list the specific evidence required for each of these priority topics.

**1) Over the next 6 months to 1 year, what are the windows of opportunity for action that might exist? (Examples include: change in government, change in ministry/ minister, new strategic plans, new reports on health system, the media focusing on certain topics, etc…)**

__________________________________________________________________________________________________________________________________________________________________________________________________________________________________________________________________________________________________________________________________________________________________________________________________________________________________________________________________________________________

______________________________________________________________________________________________________________________________________________________________

__________________________________________________________________________________________________________________________________________________________________________________________________________________________________________________________________________________________________________________________________________________________________________________________________________________________________________________________________________________________

______________________________________________________________________________________________________________________________________________________________

_______________________________________________________________________________

_____________________________________________________________________________________________________________________________________________________________________________________________________________________________________________

**2) Complete the table on the priorities for policy briefs over the next 6 months to 1 year at the national level and the specific evidence required for each of the priorities.**

| **Priority topics** | **Specific evidence needed** |
| --- | --- |
| **Example 1:** health human resources in health delivery | How can the migration of health service professionals be managed so as to reduce any negative effects on supply? What are effective retention and recruitment strategies? |
| **Example 2:** Coverage and resource allocation for the elderly | How can the state design efficient, equitable and sustainable funding systems for long-term care for older people? |
|  |  |
|  |  |
|  |  |
|  |  |
|  |  |
|  |  |
|  |  |
|  |  |
