# Supplementary material for: A multi-faceted approach to promote knowledge translation platforms in eastern Mediterranean countries: climate for evidence-informed policy
Source: Health Res Policy Syst. 2012 May 6;10:15. doi: 10.1186/1478-4505-10-15 (PMC3445832; doi:10.1186/1478-4505-10-15)
Supplement: Additional file 4 — Developing an action plan for national KT platforms. [file 1478-4505-10-15-S4.doc]

**Supplemental Material 4**

**Developing an action plan for national KT platforms**

In this group session, you are required to develop a plan of action for promoting the use of knowledge in policymaking in your country.

| **Creating KT platforms in your country building on the existing infrastructure requires:**   - - Indentifying priority themes   - Indentifying members of the core team   - Mechanisms and tools   - Planning of realistic milestones, next steps, and timelines |
| --- |

**Please follow the framework below to guide you through developing your country plan.**

1. **Develop the overall concept for the network including relevant target groups.**

________________________________________________________________________

________________________________________________________________________

________________________________________________________________________

________________________________________________________________________

________________________________________________________________________

1. **Define appropriate levels (local, provincial, national).**

________________________________________________________________________

________________________________________________________________________

1. **Identify where the network will be located.**

________________________________________________________________________

________________________________________________________________________

1. **Identify and build the core team.**

________________________________________________________________________

________________________________________________________________________

________________________________________________________________________

________________________________________________________________________

________________________________________________________________________

________________________________________________________________________

1. **Identify and initiate relevant partnerships and linkages.**

________________________________________________________________________

________________________________________________________________________

________________________________________________________________________

________________________________________________________________________

________________________________________________________________________

________________________________________________________________________

1. **Identify needed support and commitment from key stakeholders.**

________________________________________________________________________

________________________________________________________________________

________________________________________________________________________

________________________________________________________________________

________________________________________________________________________

________________________________________________________________________

1. **Identify financial support (Consider national and external sources).**

________________________________________________________________________

________________________________________________________________________

________________________________________________________________________

________________________________________________________________________

1. **Identify domains most in need of knowledge/ evidence informed decision making in your country List 3-5 priority themes.**

________________________________________________________________________

________________________________________________________________________

________________________________________________________________________

________________________________________________________________________

________________________________________________________________________

________________________________________________________________________

________________________________________________________________________

________________________________________________________________________

1. **Identify factors that contribute to insufficient use of knowledge in health policymaking (i.e. barriers).**

________________________________________________________________________

________________________________________________________________________

________________________________________________________________________

________________________________________________________________________

________________________________________________________________________

________________________________________________________________________

________________________________________________________________________

________________________________________________________________________

________________________________________________________________________

________________________________________________________________________

1. **Suggest means to better produce, disseminate and use knowledge for decision making in your country**

________________________________________________________________________

________________________________________________________________________

________________________________________________________________________

________________________________________________________________________

________________________________________________________________________

________________________________________________________________________

________________________________________________________________________

________________________________________________________________________

________________________________________________________________________

________________________________________________________________________

________________________________________________________________________

________________________________________________________________________

________________________________________________________________________

1. **Establish an approach to evaluating efforts to use knowledge in policymaking (Monitoring and Evaluation)**

________________________________________________________________________

________________________________________________________________________

________________________________________________________________________

________________________________________________________________________

________________________________________________________________________

________________________________________________________________________

________________________________________________________________________

________________________________________________________________________

________________________________________________________________________

________________________________________________________________________
